# Redescription of “*Nesticus*” *citrinus* (Taczanowski, 1874) (Araneae, Araneoidea) from French Guyana

**DOI:** 10.3897/zookeys.537.6818

**Published:** 2015-11-18

**Authors:** Yuri M. Marusik, Mikhail M. Omelko, Seppo Koponen

**Affiliations:** 1 Institute for Biological Problems of the North, Portovaya Street 18, Magadan 685000, Russia; 2 Department of Zoology & Entomology, University of the Free State, Bloemfontein 9300, South Africa; 3 Gornotaezhnaya Station FEB RAS, Gornotaezhnoe Vil., Ussuriyski Dist., Primorski krai 692533, Russia; 4 Far Eastern Federal University, Sukhanova 8, Vladivostok 690950, Russia; 5 Zoological Museum, University of Turku, FI-20014 Turku, Finland

**Keywords:** Broken-off embolus, Nesticidae, spider, Theridiidae

## Abstract

“*Nesticus*” *citrinus*, a species originally placed in *Theridion* is redescribed based on the syntype series composed by 7 females and a lectotype is designated. All syntypes have broken emboli in their epigynes. Taxonomic position of “*Nesticus*” *citrinus* is briefly discussed and its belonging to Nesticidae is doubted.

## Introduction

*Theridion citrinum* was described based on several females from Uassa in French Guyana. Original description is rather brief and lacks any drawings and comments on the epigyne. Ten years later Keyserling (1884) redescribed this species based on syntype specimens. He provided a detailed description, including leg measurements and figures of habitus and epigyne. Since then this species was only treated in two papers: by Petrunkevitch (1911) and Levi (1963). Petrunkevitch (1911) just mentioned this species in combination with currently valid genus name *Theridion*. Levi (1963) in his revision of the New World *Theridion*, transferred *Theridion citrinum* to *Nesticus*, but no arguments for this transfer were provided.

While working with collections in the Zoological Museum of the University of Turku we came upon a jar with the syntypes series of *Theridion citrinum*, that seems to have been borrowed for the revision of Nesticidae by Lehtinen and Saaristo (1980). At first glance this species looks very different from any other Nesticidae known to us, in having a whitish abdomen formed with guanine spots and lacking a comb on tarsi IV. Search of literature proved the existence of some “*Nesticus*” with white abdomen and well developed pattern in South America (Ott and Lise 2002). In addition the syntype females have a large and straight palpal claw (Fig. 3), typical for Nesticidae and lacking in Theridiidae. Examination of the epigyne reveals its unusual shape. The epigynes of three females have two thread-like arches extended over epigynal plate in the anterior part, just like it was illustrated by Keyserling (1884) and one female has one such arch. Dissection of the epigyne and its maceration showed that these arches are break-off tip of emboli.

## Material and methods

Photographs were taken using an Olympus SZX16 stereomicroscope with an Olympus E-520 camera and prepared using CombineZP software at the Zoological Museum of the University of Turku. The epigynes were dissected and macerated in 20% potassium hydroxide aqueous solution and exposed for a few minutes in an alcohol/water solution of Chlorazol Black. Length of leg segments were measured from the dorsal side. All measurements are given in millimeters.

## Taxonomy

### “Nesticus” citrinus

Taxon classificationAnimaliaAraneaeNesticidae

(Taczanowski, 1874)

Figs 1–8

Theridium citrinum : Taczanowski 1874: 57 (♀).
Theridium citrinum : Keyserling 1884: 86, pl. 4, f. 54 (♀).
Nesticus citrinus : Levi 1963: 490 (transfer of species without argumentations).

#### Types.

7♀ “Uassa-Guyane français, leg. K.Yeliki, det. H.T. Taczanowski” from Zoological Museum, Polish Academy of Sciences. Lectotype ♀ designated here, paralectotypes 3♀ and 3 juv. Most likely one syntype was taken by Keyserling (1884) who mentioned 8 specimens. Specimens were dried out and original colouration in alcohol can not be described.

#### Note.

Uassa was not located on current and old maps of the region.

#### Description.

**Figures 1–8.**
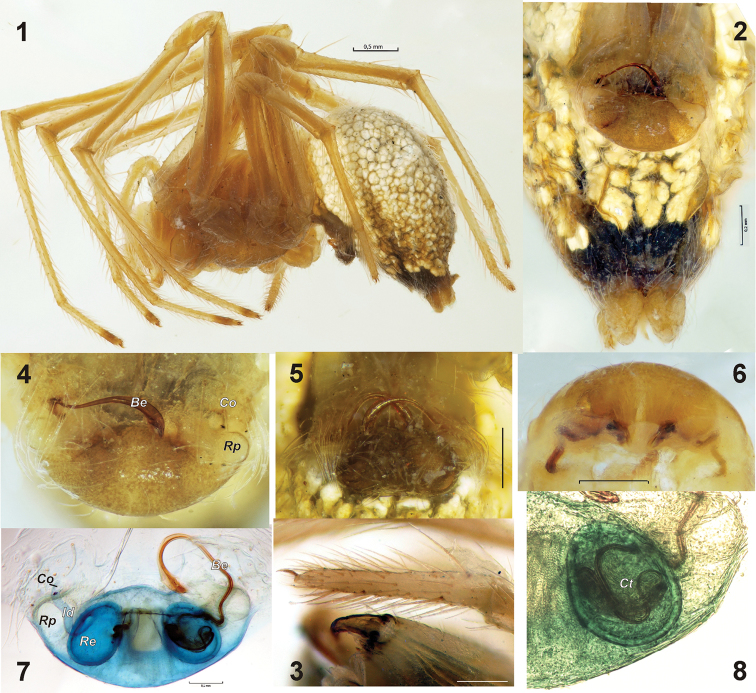
Female of *Nesticus citrinus*. **1** habitus, lateral **2** abdomen, ventral **3** palpal tarsus and chelicera **4** epigyne with one broken embolus, ventral **5** epigyne with 2 broken emboli, ventral **6** epigyne, posterior **7** epigyne after maceration, dorsal **8** receptacle with complex tip of embolus inside, dorsal. Abbreviations: *Be* broken-off terminal part of embolus, *Co* copulatory openings, *Ct* complex tip of embolus, *Id* insemination ducts, *Re* receptacle, *Rp* round pit of epigynal plate.

*Female*. Total length 3.45. Carapace length 1.6, width 1.13. According to Keyserling (1884) prosoma and appendages yellow. Tarsi of leg I‒II with dark tips (Fig. 1). Abdomen covered with white guanine spots lacking only in front of spinnerets (Figs 1–2). Palpal tarsi with large almost straight pectinate claws (Fig. 3).

Leg measurements

**Leg measurements:** 

	Femur	Patella	Tibia	Metatarsus	Tarsus	Total
I	2.03	0.75	1.83	2.03	0.85	7.49
II	2.35	0.80	1.75	2.03	0.88	7.81
III	1.50	0.53	1.00	1.20	0.55	4.78
IV	2.03	0.63	1.50	1.73	0.63	6.52

Epigyne as in Figs 4–8, large, extending epigastral fold, strongly swollen, wider than long, width subequal to 1/2 of abdomen width. Epigynal plate surrounded with long transparent hairs, its anterior edge more sclerotized than another parts. Epigynal plate with pair of round weakly sclerotized copulatory openings (*Co*), and two round pits (*Rp*) on lateral sides of the plate. Diameter of round pits more than 2 times larger than those of the copulatory openings. Copulatory openings lead to relatively short (about half of plate’s width) insemination ducts (*Id*). Receptacles (*Re*) very large, egg-shaped.

#### Comments.

All syntype females have one or two tips of embolus left (*Be*) in the epigyne (Figs 4–8). Shape and size of broken tip of emboli are the same in all observed specimens, which indicates that the embolus has a certain break-off point. The embolus penetrates not only into the insemination duct but also into the receptacle (Figs 7–8). The embolus tip (*Ct*), is quite complex and it is wider than rest of the broken part of the embolus.

Conformation of both the epigyne and tip of the embolus is unknown in other Nesticidae. This may indicate that “*Nesticus*” *citrinus* does not belong in *Nesticus* and most probably does not belong in the family Nesticidae as well.

Broken-off tip of embolus is known to occur in several families and superfamilies of spiders included in Araneoidea: Theridiidae and Linyphiidae (Wiehle 1967). “*Nesticus*” *citrinus* can not be placed in Linyphiidae due to lack of a median plate of the epigyne, and in having distinct copulatory ducts, lacking in Linyphiidae. It can neither be placed in any known Theridiidae genera, due to straight palpal claw. Bent palpal claw is known to occur in Hadrotarsinae, but it is rather short and not straight. In addition Hadrotarsinae have modified prosoma with very high clypeus, and habitually are very different from “*Nesticus*” *citrinus*.

Even though placement in *Nesticus* is somewhat doubtful, we believed it is better that it remains in *Nesticus* for now, until new specimens or a male is discovered, that could shed some light on a very mysterious spider.

## Supplementary Material

XML Treatment for “Nesticus” citrinus
